# ECG Signal Compression and Reconstruction Based on CNN-LSTM-Attention Model

**DOI:** 10.3390/s26133983

**Published:** 2026-06-23

**Authors:** Wenyan Liu, Dongzhi Chen, Ze Zhang, Yajie Cao, Yi Liu, Zhiguo Gui, Lili Liu

**Affiliations:** 1 School of Information and Communication Engineering, North University of China, Taiyuan 030051, China; 20220128@nuc.edu.cn (W.L.); 13613438102@163.com (D.C.); 15234254063@163.com (Y.C.); liuyi@nuc.edu.cn (Y.L.); gzgtg@163.com (Z.G.); 2 School of New Energy, Inner Mongolia University of Technology, Ordos 017010, China; zhangze210@imut.edu.cn

**Keywords:** ECG signal, compressive sensing, convolutional neural network, long short-term memory network, attention mechanism

## Abstract

The high prevalence of cardiovascular diseases and the extensive application wearable electrocardiogram (ECG) devices for long-term monitoring have posed significant challenges for the transmission, storage, and real-time processing of massive amounts of ECG data. Consequently, efficient ECG compression and reconstruction have become a research priority in remote ECG monitoring. Traditional compressed sensing is complex and has high computational overhead, while single deep learning models cannot simultaneously extract local waveforms and model temporal dependencies. To address these shortcomings in the reconstruction process, this paper presents a CNN-LSTM-Attention hybrid model. This model utilizes a convolutional neural network (CNN) to capture local ECG waveform features, employs a long short-term memory (LSTM) network to learn long-term temporal dependencies, and introduces an attention mechanism to weight and fuse key diagnostic features, enabling accurate focus on key components including the QRS complex and ST segment. Experimental results on the MIT-BIH Arrhythmia dataset demonstrate that across the full compression range of 0.1–0.9, the proposed model achieves favorable comprehensive performance. Its PRD is stabilized at 10–12%, the SNR stays above 20 dB, and the RMSE is mostly lower than 0.25 mV. In terms of reconstruction accuracy and stability, our model outperforms the single CNN and CNN-LSTM models by a large margin.

## 1. Introduction

As a core physiological signal reflecting cardiac electrical activity, the ECG can accurately capture key waveform features such as the P wave, QRS complex, ST segment, and T wave and serves as the gold standard for the clinical diagnosis of cardiac diseases such as arrhythmias and myocardial ischemia [1,2]. With the widespread adoption of wearable ECG devices and the rapid development of telemedicine technologies, long-term continuous monitoring has become a reality. The resulting massive volume of ECG data poses significant challenges for terminal storage, wireless transmission bandwidth, and device computing power [3,4,5]. How to achieve efficient compression and high-precision reconstruction while ensuring the clinical diagnostic value of ECG signals has become a key technical bottleneck hindering the large-scale deployment of remote ECG monitoring systems, attracting widespread attention from both academia and industry.

Early ECG signal compression and reconstruction primarily relied on traditional compressive sensing methods [6,7,8]. These methods are based on signal sparsity and achieve signal compression and recovery via wavelet transforms, dictionary learning, greedy algorithms, or convex optimization. Although traditional methods can achieve certain reconstruction results at low compression ratios, they have significant drawbacks: the reconstruction process is complex, and iterative computations have high computational overhead; at high compression ratios, low-amplitude fine waveforms are easily lost, making it difficult to preserve key pathological features; and they heavily rely on manually designed transform bases and observation matrices, resulting in insufficient generalization ability and robustness, and are difficult to adapt to the real-time processing requirements of wearable devices.

In recent years, deep learning has become the mainstream approach in ECG signal compression and reconstruction due to its advantages, including end-to-end learning, adaptive feature extraction, and fast inference.

CNNs can effectively extract local waveform features of signals and well characterize morphological patterns of ECG signals, including the QRS complex and ST segment. Mangia et al. [9] estimated confidence scores using a CNN predictor trained together with a decoder via linear mapping. The adoption of short windows can greatly reduce computational complexity. Shrivastwa et al. [10] proposed a CNN-based compressed sensing (CS) framework for compression and reconstruction of biosignals. The above studies verify that CNNs have excellent applicability in the field of compression and reconstruction of biological signals. As a classic network for local feature learning, CNNs can stably retain core waveform features of ECG signals that are closely related to clinical diagnosis and easily meet the real-time processing demands of wearable ECG monitoring devices in practical applications.

ECG signals are time-series signals, and standard CNNs lack the ability to model temporal relationships, making it difficult to capture long-range dependencies in signals. LSTM [11] is widely adopted to model time-series data for ECG processing tasks. As an improved variant of Recurrent Neural Networks (RNNs), LSTM is well suited for processing long-sequence signals. It alleviates the long-range dependency and vanishing gradient and exploding gradient issues of RNNs and effectively compensates for the inherent shortcomings of CNNs in long-sequence modeling.

To combine the strengths of both networks, researchers have proposed hybrid CNN-LSTM models, which balance local features and temporal information to a certain extent. Zhang et al. [11] proposed a non-iterative fast reconstruction algorithm CSNet integrating CNN and LSTM. This method outperforms traditional reconstruction algorithms in signal reconstruction quality and speed.

However, the memory mechanism of LSTM leads to serial computation, and low training efficiency has long been a drawback. Novel neural network architectures are urgently needed to support parallel computation while maintaining positional memory capabilities. The adoption of attention mechanisms has greatly improved model performance. When processing sequences or structured data, attention mechanisms enable networks to dynamically adjust attention on different information segments. In ECG signal processing, the model can focus on frequency bands critical to ECG morphology, such as the low-frequency energy of P waves and QRS complexes.

To address these issues, this paper introduces an attention mechanism into the CNN-LSTM hybrid architecture, proposing a deep learning reconstruction model, CNN-LSTM-Attention. This model fully integrates the local waveform extraction capability of CNNs, the long-term temporal dependency modeling capability of LSTM, and the adaptive weighting and focusing capability of the attention mechanism on key diagnostic features, thereby achieving high-precision and high-stability reconstruction of ECG signals at high compression ratios. The main contributions of this paper are as follows: by incorporating the attention mechanism to construct a CNN-LSTM-Attention hybrid reconstruction model, we combine the local feature extraction capabilities of CNNs with the long-term temporal modeling advantages of LSTM. Furthermore, through the attention mechanism, we achieve adaptive enhancement of key features, effectively improving the accuracy and stability of ECG signal reconstruction. We designed ablation experiments and conducted multi-model comparisons, validating the model on public ECG datasets. The model outperformed comparison models in both reconstruction accuracy and waveform integrity, demonstrating the attention mechanism’s role in improving reconstruction accuracy at high compression ratios. We conducted a combined quantitative and qualitative analysis of the experimental results, objectively evaluating model performance using quantitative metrics such as Root Mean Square Error (RMSE), PRD, and SNR while also verifying the authenticity and completeness of the reconstruction results through intuitive waveform comparisons.

It should be emphasized that this work is a preliminary investigation into the feasibility of ECG signal reconstruction using hybrid deep learning architectures under extreme compression conditions. The system performs end-to-end reconstruction at high compression ratios (CR = 0.1–0.9), meaning only 10–90% of Discrete Cosine Transform (DCT) coefficients are preserved. Restricted by the network scale and parameter quantity, the achieved reconstruction performance is relatively limited: the PRD ranges from approximately 34% to 35%, and the SNR is around 9 dB, which is far below the clinical diagnostic requirement (PRD < 10%).

We do not intend to claim that the proposed model can be directly applied to clinical diagnosis or wearable medical monitoring scenarios. Instead, this study mainly verifies the applicability of the CNN-LSTM-Attention architecture and the effectiveness of the attention mechanism for ECG compression and reconstruction and provides a baseline for further performance improvement.

## 2. Related Work

### 2.1. Traditional ECG Signal Compression and Reconstruction Methods

Traditional methods focus on signal sparsity, compress signal via observation matrices, and recover them with dedicated algorithms. Generally, traditional sparse signal reconstruction methods can be categorized into four main types: convex relaxation optimization methods, greedy matching pursuit, Bayesian inference methods, and combinatorial optimization methods. Daponte et al. [11] decomposed multi-lead ECG signals using a Mexican hat wavelet matrix to construct a deterministic observation matrix for compression. With the evolution of compressive sensing, data-driven learning dictionaries have gradually replaced fixed transformation bases. Researchers trained ECG-oriented dictionaries using the K-SVD algorithm to adapt to irregular ECG signals. Surekha et al. [12] proposed a joint wavelet-compressed sensing framework: the signal is first sparsely decomposed using the discrete wavelet transform and then compressed via an observation matrix and Huffman coding. At the receiver end, decoding and observation recovery are performed first, followed by signal reconstruction using a block-sparse Bayesian learning algorithm with boundary optimization. Abhishek et al. [13] designed a set of biorthogonal wavelet filters and applied them to the sparse decomposition of signals. They used multiple observation matrices to compress the signal and, in the reconstruction stage, employed a boundary-optimized block-sparse Bayesian learning algorithm. Zhang et al. [14] proposed an ECG signal reconstruction method that integrates adaptive dictionaries with matched filtering. The core idea is constructing multiple overcomplete dictionaries based on the positions of R-wave peaks in the original ECG signal. Simultaneously, matched filtering is used to precisely locate the R-wave peaks in the compressed signal, and the signal is ultimately reconstructed by matching them with the corresponding overcomplete dictionaries. Thanks to the high compatibility of the constructed overcomplete dictionaries with the inherent characteristics of ECG data, the signal reconstruction quality of this method has been further improved.

Although traditional methods can achieve a certain degree of compression and reconstruction—reaching low PRD values at specific compression ratios—they have inherent drawbacks: low-amplitude signal components are prone to loss at high compression ratios, and reconstruction errors increase significantly; furthermore, reconstruction algorithms often rely on iterative computations, resulting in high complexity and long processing times. In recent years, compression sensing methods incorporating deep learning have gradually become a hot topic of research.

### 2.2. Applications of Deep Learning in ECG Signal Compression and Reconstruction

Deep learning techniques realize joint optimization of compression and reconstruction through end-to-end learning, removes complex sparse decomposition steps. They possess key strengths such as high reconstruction accuracy, fast processing speeds, and adaptability to signal distributions. Tang et al. [7] proposed a multi-task model that integrates CS with CNNs. This model employs bias-free fully connected layers to perform signal compression and, through shared layers and two task branches, simultaneously achieves signal reconstruction and classification. Prono et al. [11] proposed a signal compression and reconstruction model based on deep neural networks (DNNs), which consists of two core modules: an encoding layer responsible for signal compression and a decoding layer responsible for signal reconstruction; Hua et al. [15] proposed a method for ECG signal compressive sensing that integrates improved Inception blocks with LSTM. This method employs three consecutive convolutional layers to perform signal compression: first, the signal dimensions are restored through an initial reconstruction, followed by a secondary reconstruction using the improved Inception block and LSTM to further enhance reconstruction quality. Zhang et al. [16] first compress the signal using an observation matrix and then perform a transposed projection on the compressed signal; subsequently, they use a CNN and an LSTM network to learn the mapping relationship between the transposed projection signal and the original signal. This method not only effectively cuts down reconstruction time but also lowers reconstruction error.

In recent years, Lal et al. [17] presented an end-to-end compression and reconstruction framework using data-driven sensing matrices and lightweight neural networks. This method jointly learns adaptive sensing schemes and hardware-friendly reconstruction modules and achieves notable accuracy gains over conventional CS methods on the MIT-BIH dataset. As a model-data dual-driven method, PC-BCSNet combines pattern-coupled sparse Bayesian learning with data-driven deep learning to build an interpretable deep iterative inference network. It surpasses state-of-the-art algorithms in both reconstruction accuracy and inference speed [18]. CEDRC-Net integrates a Transformer-based convolutional denoising autoencoder and a temporal fusion Transformer for signal recovery. On the MIMIC-IV dataset, this network attains a low mean absolute error (MAE) of 0.015 [19].

Although the aforementioned deep learning models have achieved certain progress—with the CNN-LSTM model balancing local feature extraction and temporal dependency modeling to a certain degree—they still exhibit significant shortcomings in reconstructing ECG signals at high compression ratios. Specifically, the model assigns equal weights to both local waveforms and temporal features, leading to issues such as the weakening of critical information at high compression rates.

To further strengthen the model’s perception on key features, researchers have begun introducing attention mechanisms into ECG signal processing to enhance the model’s capability to perceive and utilize critical information. The attention mechanism can adaptively assign higher weights to key features in ECG signals, enabling the model to automatically focus on clinically significant regions such as the QRS complex, P wave, and T wave when processing long-term time-series data. This effectively suppresses redundant information and noise interference, overcoming the limitation of traditional networks in paying insufficient attention to subtle features. Therefore, this paper proposes a hybrid network model based on CNN-LSTM-Attention. This model fully integrates the local feature extraction capabilities of convolutional neural networks, the temporal dependency modeling capabilities of long short-term memory networks, and the adaptive weighting of key features via attention mechanisms. It can accurately preserve signal details even at high compression ratios, improving the model’s feature utilization and robustness, delivers more stable reconstruction results, and can better satisfy clinical application requirements.

## 3. Methodology

**Figure 1:**
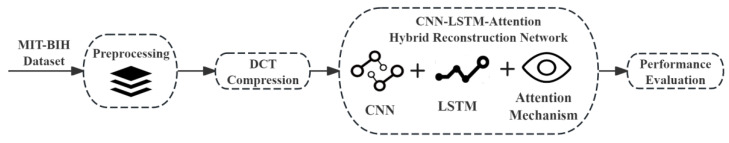
Overall schematic of the research protocol.

The overall research framework of this paper is illustrated in Figure 1, which consists of four modules: data preprocessing; DCT compression; a CNN-LSTM-Attention hybrid reconstruction network; and performance evaluation. DCT is employed for signal dimensionality reduction. The CNN is responsible for accurately capturing local waveform features of the ECG signal, the LSTM is used to extract long-term temporal dependencies of the signal, and the attention mechanism conducts weighted fusion of the extracted features. This approach highlights key diagnostic components such as the QRS complex and ST segment, thereby enhancing the clinical applicability of the reconstructed signal while maintaining a high compression ratio.

### 3.1. Data Preprocessing

When preprocessing the three datasets (Train.csv, Validation.csv, and Test.csv), baseline wander correction is first applied to the raw ECG signals to remove low-frequency interference. Subsequently, a sliding window strategy is employed to segment the ECG signals, where each window contains 5 data points. Every five consecutive rows of raw data (each with N features) are reshaped into one row with 5N features. To maintain data consistency, any remaining samples that cannot be fully divided by the window length are discarded. Finally, the processed datasets are converted into 2D matrix format, thereby compressing the original ECG sequences.

### 3.2. DCT Compression

The preprocessed single-segment ECG signal is ϵ RN, where N is the signal length. Its DCT transform is defined as:

(1) $$Y(k)\;=\sqrt{\frac{2}{N}}a(k)\sum_{t=0}^{N-1}x(t)\cos(\frac{(2t+1)k\Pi }{2N}),\;k\;=\;0,1,...,N-1$$

where a(0) = 1, and α(k) =$\;\sqrt{2}$ (k ≥ 1).

Through the DCT transformation, most energy in the ECG signal is concentrated in a small number of low-order transform coefficients, while high-order coefficients primarily correspond to noise and redundant information. Therefore, this paper achieves signal compression by retaining the first M low-frequency coefficients and eliminates the rest of the high-order coefficients. The compression ratio (CR) is defined as:

(2) $$\mathrm{CR}\;=\;\frac{N}{M}$$

ECG signals exhibit typical sparsity, with their energy highly concentrated in the low-frequency band. By leveraging the energy concentration property of the DCT, the preprocessed ECG signal is transformed to retain low-frequency coefficients that carry major signal energy and discard high-frequency redundant coefficients, thereby achieving compression. This step yields low-dimensional inputs for the subsequent reconstruction network.

### 3.3. CNN-LSTM-Attention Hybrid Reconstruction Network

**Figure 2:**
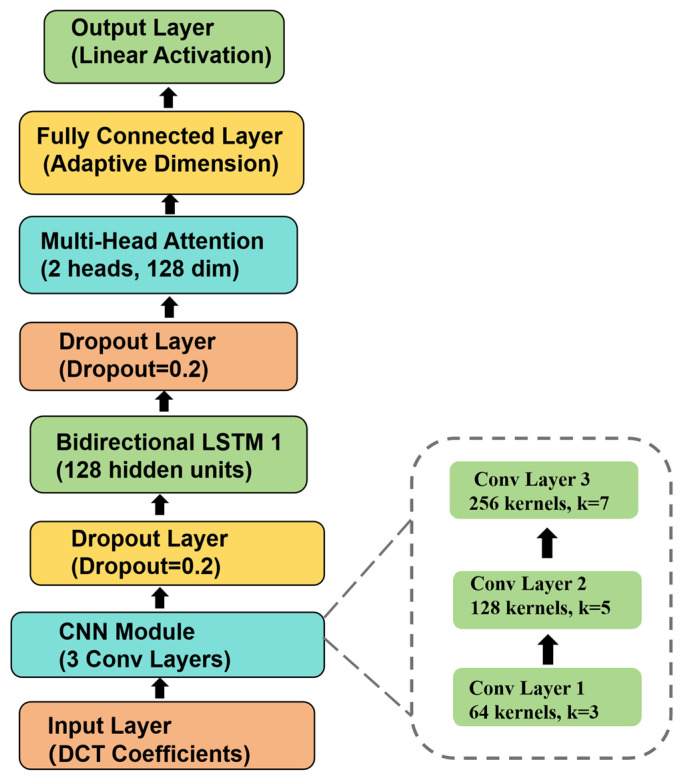
Network architecture of the CNN-LSTM-Attention model.

A hybrid reconstruction model combining CNN, LSTM, and Attention maps compressed DCT coefficients to ECG signals in the original dimension to achieve high-precision reconstruction. Figure 2 illustrates the overall network architecture, and Table 1 details network parameters.

The CNN module adopts a three-layer 1D convolutional neural network architecture, specifically designed for the feature extraction requirements of one-dimensional time-series signals, such as ECG and vibration. The network consists of three convolutional layers cascaded. The first layer has 64 convolutional kernels with a size of 3, used to extract shallow local features of the signal; the second layer has 128 convolutional kernels with a size of 5, used to capture the signal’s mesoscale dependencies deep correlation features. The third layer has 256 convolutional kernels with a size of 7, used to further integrate global temporal information and achieve a comprehensive feature representation. After each convolutional operation, a ReLU activation function is applied to perform nonlinear mapping, strengthening the network’s capability to fit complex temporal patterns.

The LSTM module uses a bidirectional LSTM architecture with two stacked layers. The first layer is configured with 128 units, enabling it to output the full time series and preserving the forward and backward temporal dependencies, thereby providing sufficient feature information for subsequent layers. This layer is followed by a Dropout layer with a dropout rate of 0.2 to mitigate overfitting. The second layer consists of 64 units and outputs only the feature vector at the final time step, aggregating the temporal information across the entire sequence into a global, fixed-dimensional feature representation. This layer is followed by a Dropout layer (with a dropout rate of 0.2) to improve model generalization. This two-layer bidirectional LSTM architecture enables the model to learn complex temporal patterns in the signal at different scales. Within the LSTM cells, data sequentially passes through the forget gate, input gate, and output gate, enabling selective retention and updating of information. Model parameters are iteratively updated via the backpropagation through time until the loss function gradually converges. The Adam optimizer is selected, with an initial learning rate set to 0.0005, a batch size of 16, and a maximum of 10 training epochs. An early stopping strategy is applied: the training process will be terminated, and the optimal model weights will be loaded if the validation loss does not decrease for five consecutive epochs. Following the LSTM layer, a fully connected layer maps the extracted temporal features back to the original signal dimensions to generate the reconstructed signal sequence.

The attention mechanism module adopts a multi-head self-attention structure based on scaled dot products, which belongs to the soft self-attention category. It is connected after the bidirectional LSTM layer to further strengthen the modeling of long-range temporal feature correlations. This module maps the same temporal features output by the LSTM into three groups of learnable vectors: Query, Key, and Value. Through two parallel attention heads, it computes the similarity between temporal features across different subspaces, quantifies feature dependencies using a scaled dot-product function, and generates continuously differentiable attention weights via the Softmax function, thereby achieving focus on key temporal features and suppression of redundant information.

It is worth noting that the DCT converts ECG signals into the frequency domain and disrupts the original temporal order. Accordingly, the subsequent LSTM module does not model temporal sequences of raw ECG signals but captures the intrinsic dependencies among frequency-domain coefficients sorted by frequency indices. DCT coefficients are arranged in ascending order of frequency, where low-frequency coefficients contain most of the signal energy, while high-frequency coefficients tend to approach zero, presenting a smooth attenuation pattern between adjacent coefficients. The gating mechanism of LSTM is well suited to capture such long-range correlations within numerical coefficient sequences.

In this context, the sequential arrangement of DCT coefficients corresponds physically to an incremental frequency order from low to high. The correlations between adjacent coefficients further reflect the inherent compactness and smoothness constraints of energy distribution in ECG signals. Moreover, no explicit inverse DCT is adopted for frequency-to-time domain conversion in this study. Instead, the output layer of the proposed CNN-LSTM-Attention reconstruction network (a fully connected layer with linear activation) implicitly implements domain transformation via end-to-end learning. Specifically, the network directly regresses complete time-domain ECG waveforms from compressed DCT coefficients, where the dimension of the output layer is strictly matched to the length of the original ECG signal, enabling high-precision signal reconstruction.

Overall, feeding DCT coefficient sequences into LSTM networks is mathematically grounded and physically interpretable. This technical paradigm proves to be a reasonable and effective solution for the interdisciplinary integration of compressed sensing and deep learning in ECG signal reconstruction.

## 4. Experimental Validations

This chapter provides a comprehensive evaluation and analysis of the signal reconstruction performance of the CNN-LSTM-Attention model. Using quantitative metrics and visualization results, we validate the superiority of the experimental model and investigate how varying compression rates affect its reconstruction performance.

### 4.1. Dataset

This paper employs the MIT-BIH Arrhythmia Dataset for experimental evaluation. It comprises 48 ambulatory ECG recordings acquired at a sampling rate of 360 Hz, mainly using the MLII lead. The dataset includes normal sinus rhythms and 14 common arrhythmia categories, providing high data diversity and reliable clinical representativeness for performance evaluation.

This paper randomly splits the 48 ECG recordings for experimental evaluation. Specifically, 80% of the total data is allocated to the training and test subsets, which are further divided at an 8:2 ratio. The remaining 20% of the data is independently reserved as the validation set.

### 4.2. Evaluation Metrics

To comprehensively validate the overall performance of the CNN-LSTM-Attention hybrid reconstruction network in ECG signal reconstruction tasks, the Percentage Root Mean Square Error (PRD), Root Mean Square Error (RMSE), and Signal-to-Noise Ratio (SNR) are selected as core evaluation metrics, defined as follows:

(1) PRD:

The Percentage Root Mean Square Error measures the deviation between the reconstructed and original signals; a smaller value indicates higher reconstruction accuracy. Its calculation formula is:

(3) $$\mathrm{PRD}\;=\frac{\;\sqrt{\sum_{i=1}^{N}{\left(x_{i}-\hat{x_{i}}\right)}^{2}}}{\sqrt{\sum_{i=1}^{N}{\hat{x_{i}}}^{2}}}\times 100\%$$

where x_i_ represents the original ECG signal, $\hat{x_{i}\;}$ represents the model-reconstructed signal, and N denotes the signal length.

(2) RMSE:

This is used to measure the global error between the reconstructed signal and the original signal; a smaller value indicates less error. The formula is:

(4) $$\mathrm{RMSE}=\sqrt{\frac{1}{N}\sum_{i=1}^{N}{\left(x_{i}-\hat{x_{i}}\right)}^{2}}$$

(3) SNR:

This is used to measure the ratio of useful signal to noise, reflecting signal fidelity; a higher value indicates better reconstruction quality. The formula is:

(5) $$\mathrm{SNR}\;=\;10\;\log_{10}\left(\frac{\sum_{i=1}^{N}{\hat{x_{i}}}^{2}}{\sum_{i=1}^{N}{\left(x_{i}-\hat{x_{i}}\right)}^{2}}\right)$$

Through comprehensive calculation and analysis of multidimensional metrics, we systematically evaluate the model’s ECG signal reconstruction performance across different compression ratios, verify its ability to extract local waveforms and long-term temporal features from ECG signals, and assess the effectiveness of the attention mechanism in focusing on core diagnostic features.

(4) Loss function:

The primary loss function used during training is the Mean Squared Error (MSE). The mathematical expression is as follows:

(6) $$L_{MSE}=\frac{1}{N}\sum_{i=1}^{N}{\left({\hat{x_{i}}-x}_{i}\right)}^{2}$$

where N denotes the signal length, $x_{i}$ represents the original time-domain ECG signal, and ${\hat{x}}_{i}$ stands for the reconstructed time-domain signal generated by the model.

### 4.3. Ablation Studies

To validate the necessity of each module in the proposed hybrid network, we designed ablation comparison experiments. CNN model: This retains only one-dimensional convolutional modules, without LSTM or temporal modeling capabilities; CNN-LSTM model: This retains CNN + bidirectional LSTM, without the attention mechanism; CNN-LSTM-Attention model: This is the complete model proposed in this paper, including CNN, bidirectional LSTM, and multi-head self-attention.

All models use the same dataset, DCT compression strategy, and training parameters; only the network architecture differs, ensuring experimental fairness.

### 4.4. Comparison of Results

**Figure 3:**
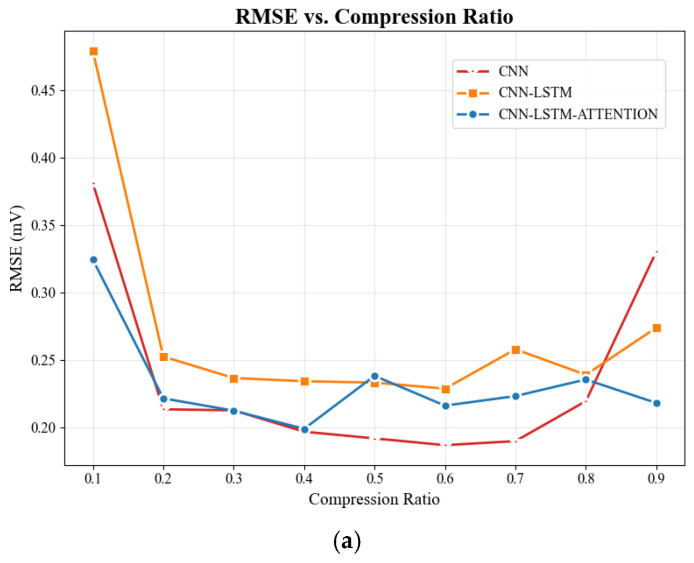

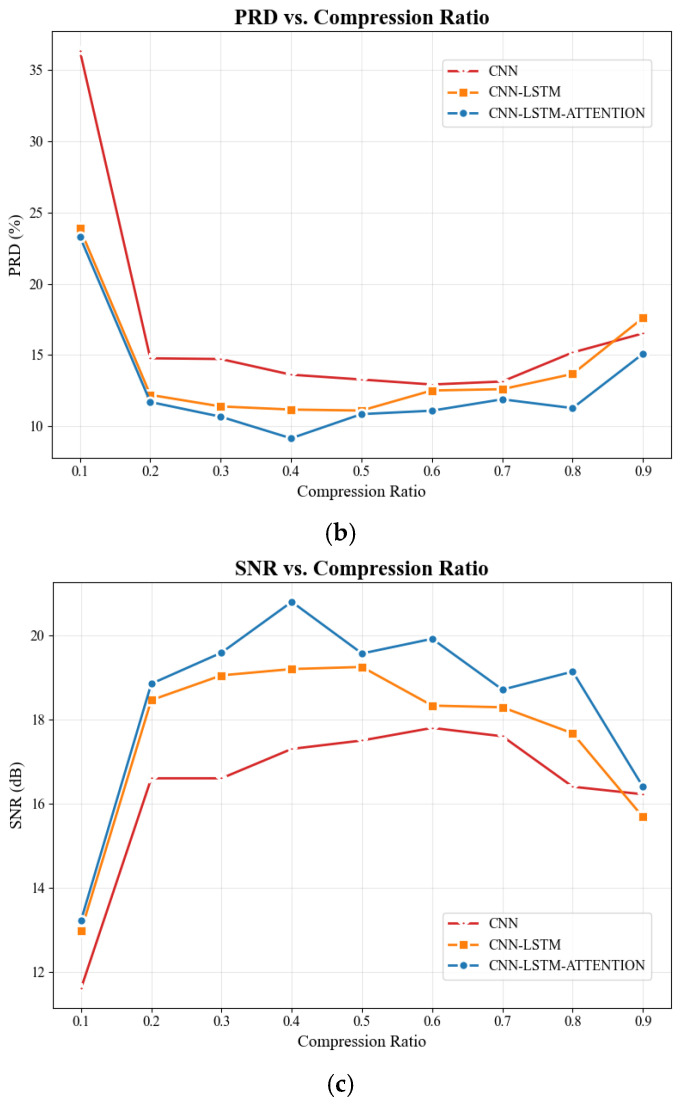
Comparison of model performance: (**a**) RMSE comparison results, (**b**) PRD comparison results, and (**c**) SNR comparison results.

The reconstruction performance of the three models (CNN, CNN-LSTM, and CNN-LSTM-Attention) was analyzed at compression ratios from 0.1 to 0.9. As shown in Table 2, four metrics are used for evaluation: RMSE, PRD, SNR, and correlation coefficient. Figure 3 shows the performance comparison of the three models in terms of RMSE, PRD and SNR.

The comparison results indicate significant performance differences among the three models: the CNN model exhibits poor reconstruction quality, with the PRD ranging from 15% to 36% and the SNR below 18 dB. The CNN-LSTM model shows improved performance, with the PRD reduced to 12–24% and the SNR reaching up to 19.2 dB. Meanwhile, the CNN-LSTM-Attention model maintains the lowest PRD (stabilized at 10–12% in the middle compression range), the highest SNR (peaking above 20 dB), and the smallest RMSE (mostly below 0.25 mV) across all compression rates, demonstrating the best reconstruction performance and high stability, thereby validating the superiority of the model used in this experiment.

**Figure 4:**
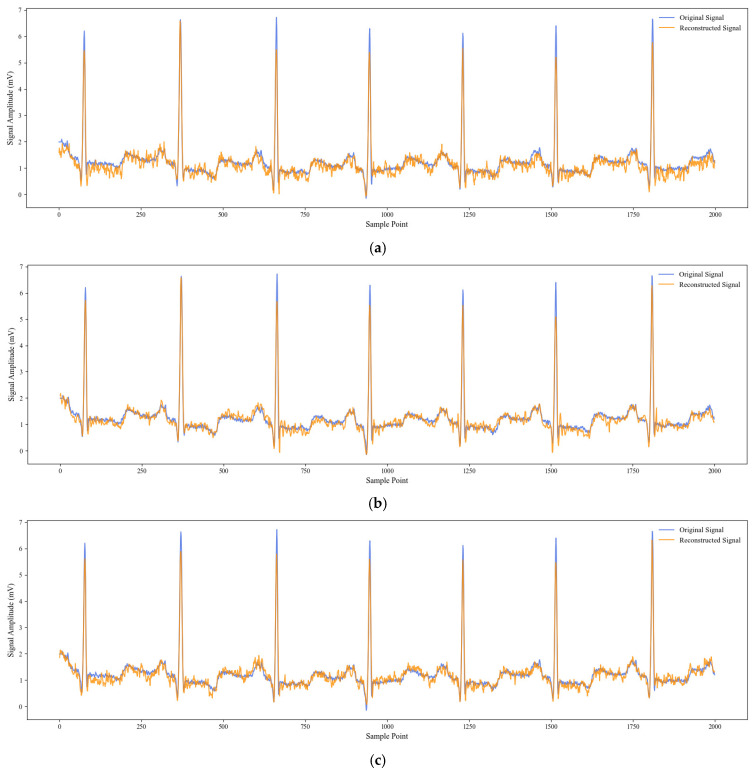
Comparison of waveforms between the original signal and the reconstructed signal at a compression ratio of 0.4: (**a**) results of the CNN model, (**b**) results of the CNN-LSTM model, and (**c**) results of the CNN-LSTM-Attention model.

**Figure 5:**
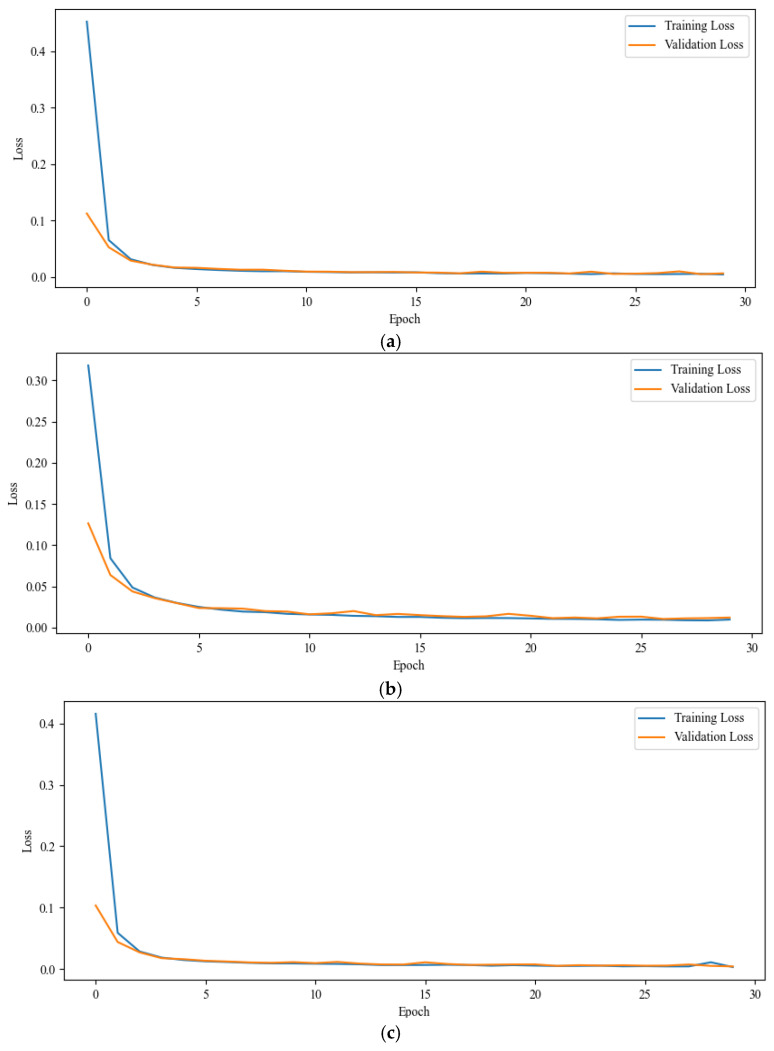
Evolution of training and validation losses for the model at a compression ratio of 0.4: (**a**) results of the CNN model, (**b**) results of the CNN-LSTM model, and (**c**) results of the CNN-LSTM-Attention model.

**Figure 6:**
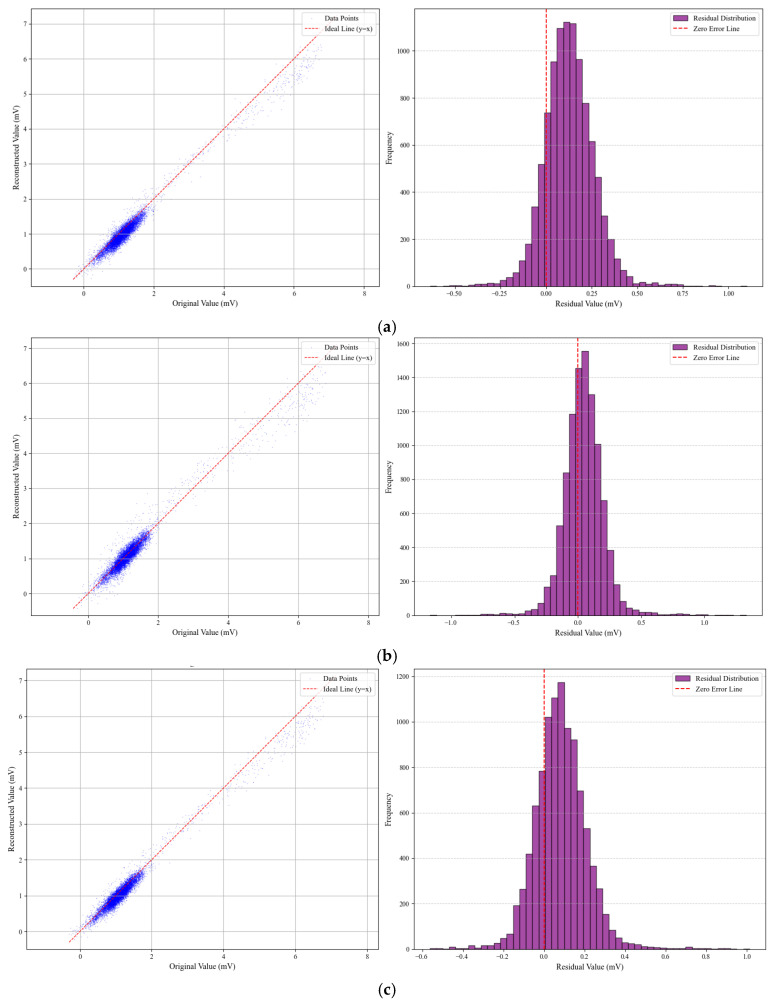
Scatter plots and residual distribution plots of the model’s original values and predicted values at a compression ratio of 0.4: (**a**) results of the CNN model, (**b**) results of the CNN-LSTM model, and (**c**) results of the CNN-LSTM-Attention model.

Figure 4 compares the waveforms between the original signal and the reconstructed signal at a compression ratio of 0.4. Figure 5 shows the evolution of training and validation losses for the three models at a compression ratio of 0.4. Figure 6 presents scatter plots and residual distribution plots of the model’s original values and predicted values at a compression ratio of 0.4.

The ECG signals used in this study were preprocessed with a linear scaling factor to fit the input range of the deep learning model, resulting in an amplitude range of approximately 0 to 7 mV. The scaling operation preserves the relative morphological features of the original physiological signals and does not affect the performance evaluation of the compression and reconstruction model.

A comparison reveals that the CNN model exhibits high training loss, with scatter points deviating from the ideal line and a wide distribution of residuals; the reconstructed signal waveform shows a significant discrepancy from the original signal waveform. The CNN-LSTM model exhibits lower training loss, with data points closely aligned with the ideal line and a narrower residual distribution, successfully reconstructing the signal waveform to a large extent; however, the reconstructed waveform still suffers from blurred details. The CNN-LSTM-Attention model has the lowest loss, with data points closely aligned with the ideal line and a narrow residual distribution. The reconstructed signal waveform maintains the original signal’s overall rhythm, yielding the best results.

### 4.5. Comparison with Traditional CS Methods

To further verify the effectiveness and superiority of the proposed CNN-LSTM-Attention framework, we select two representative traditional compressed sensing algorithms, namely Orthogonal Matching Pursuit (OMP) and Basis Pursuit (BP), to conduct quantitative comparison experiments under different compression ratios on the MIT-BIH Arrhythmia dataset. Three core metrics, including PRD, SNR, and RMSE, are adopted to evaluate the reconstruction performance.

The comparison results are presented in Table 3. At low compression ratios (CR = 0.1, 0.2), the traditional OMP and BP algorithms show limited reconstruction performance, with high PRD and RMSE values as well as low SNR. Although their reconstruction quality gradually improves with the increase of compression ratio, it is still inferior to the proposed method overall. In contrast, our CNN-LSTM-Attention model outperforms the two traditional CS algorithms significantly across all compression ratios, achieving lower PRD, smaller RMSE and higher SNR.

## 5. Discussion

Although the method proposed in this study has achieved certain results, it still has many shortcomings and significant room for improvement. Future research could focus on the following areas:

This study utilized only the MIT-BIH single-lead dataset, resulting in a relatively limited range of data types and scenarios. Future work could incorporate multi-lead ECG datasets or clinical ECG data from different age groups and medical conditions to enhance the model’s generalization ability.

The MIT-BIH dataset used in this study is standardized and contains minimal noise interference. However, in real-world scenarios, signals collected by wearable devices are prone to interference from human movement and environmental electromagnetic fields. The model’s performance in such complex noise environments has not been tested, and its effectiveness in practical applications may be uncertain. Therefore, future research could train and validate the model using real-world datasets containing diverse noise types or simulated datasets with added noise to enhance its adaptability in practical scenarios.

Furthermore, the current model still cannot meet the requirements for clinical diagnosis, with its reconstruction performance standing at a PRD of roughly 10–12% and an SNR of over 20 dB, while the clinical standard requires PRD below 10%. This study mainly verifies the applicability of hybrid deep learning architectures under extreme compression scenarios, and we do not claim that the proposed method can be directly applied in clinical practice. In our follow-up research, we will further optimize the network structure, adopt perceptual loss functions and adversarial training strategies to lower the PRD to less than 10%.

This paper randomly splits all 48 ECG recordings in the MIT-BIH Arrhythmia Dataset as a whole: 80% of the total data is divided into training set and test set at a ratio of 8:2, and the remaining 20% of the data is used as an independent validation set. It should be noted that this division method is based on overall random segmentation rather than inter-subject independent segmentation. This division mode may lead to the existence of homologous data from the same subject in the training set, test set, and validation set, which will make the model’s evaluation results overly optimistic to a certain extent and limit the generalization ability of the model to unknown subjects. In our follow-up research, we will adopt the inter-subject dataset division method to further verify the practical performance of the model.

## 6. Conclusions

This experiment addresses the signal compression and reconstruction requirements for remote ECG monitoring. To overcome the low reconstruction efficiency of traditional compressed sensing methods and the difficulty of single deep learning models in balancing both local and temporal features of ECG signals, a hybrid network reconstruction model based on CNN-LSTM-Attention was designed. The main contributions are as follows:

We conducted a theoretical study of the attention mechanism, analyzing the shortcomings of traditional compressed sensing methods—namely, their complex iterative computations—and the incomplete feature extraction of single deep learning models, thereby providing theoretical support and a design direction for the construction of the hybrid network model.

In terms of model performance, the superiority arises not from a single component but from the synergistic effect of CNN, LSTM, and attention mechanisms. At low compression ratios, DCT enables preliminary signal compression yet inevitably loses partial high-frequency details. Herein, the CNN module excels at local feature extraction and effectively recovers morphological features of key waveforms including the QRS complex and ST segment, laying a solid foundation for subsequent reconstruction. Meanwhile, the LSTM module captures long-range dependencies within ECG signals and tracks temporal rhythm changes, which prevents rhythm distortion that commonly occurs when using CNN alone. The adopted multi-head self-attention mechanism further assigns adaptive weights to clinically significant features. During reconstruction, the model automatically focuses on waveform segments critical for diagnosis and suppresses noise and redundant interference. As a result, favorable and stable reconstruction performance is maintained across various compression ratios.

The comparative experimental results clearly verify this point. The performance metrics of the CNN, CNN-LSTM, and CNN-LSTM-Attention models were compared over a compression ratio range of 0.1–0.9, and both the comparison and reconstruction results were visualized.

The experimental results show that the proposed CNN-LSTM-Attention model maintains a stable PRD of 10–12% and a high SNR above 20 dB across the entire compression range, accompanied by consistently low RMSE values. The proposed method effectively compensates for the insufficient fine-grained feature extraction of pure CNN and CNN-LSTM models, achieving prominent improvements in reconstruction accuracy, waveform fidelity, and overall stability under varying compression conditions.

## Figures and Tables

**Table 1:** Parameter settings for the CNN-LSTM-Attention hybrid reconstruction network.

Module	Layer Name	Parameter Configuration
Input Module	Input Layer	Dimension: length of DCT coefficients after compression
CNN Module	Convolution Layer 1	64 kernels, kernel size 3, ReLU activation
	Convolution Layer 2	128 kernels, kernel size 5, ReLU activation
	Convolution Layer 3	256 kernels, kernel size 7, ReLU activation
LSTM Module	Bidirectional LSTM Layer 1	Set to 128 hidden units
	Dropout Layer	Dropout rate 0.2
	Bidirectional LSTM Layer 2	Set to 64 hidden units
	Dropout layer	Dropout rate 0.2
Attention Mechanism Module	Multi-head Attention	Embedding dimension 128, head count 2, scaled dot-product attention
Fully Connected and Output Modules	Fully Connected Layer	Neuron adaptive dimension, ReLU activation
	Output Layer	Linear activation

**Table 2:** Comparison of reconstruction effects of three models under compression ratios from 0.1 to 0.9.

Compression Ratio	Model	RMSE (mv)	PRD (%)	SNR (dB)	Correlation Coefficient
0.1	CNN	0.3811	36.36	11.60	0.9684
	CNN-LSTM	0.4793	23.88	12.97	0.9690
	CNN-LSTM-Attention	0.3244	23.3	13.22	0.9787
0.2	CNN	0.2134	14.76	16.60	0.9801
	CNN-LSTM	0.2525	12.19	18.46	0.9826
	CNN-LSTM-Attention	0.2215	11.69	18.85	0.9855
0.3	CNN	0.2126	14.71	16.60	0.9802
	CNN-LSTM	0.2366	11.38	19.05	0.9835
	CNN-LSTM-Attention	0.2124	10.66	19.59	0.9861
0.4	CNN	0.1968	13.61	17.30	0.9813
	CNN-LSTM	0.2342	11.16	19.20	0.9837
	CNN-LSTM-Attention	0.1989	9.14	20.80	0.9870
0.5	CNN	0.1918	13.27	17.50	0.9816
	CNN-LSTM	0.2332	11.09	19.25	0.9837
	CNN-LSTM-Attention	0.2382	10.84	19.57	0.9844
0.6	CNN	0.1868	12.92	17.80	0.9820
	CNN-LSTM	0.2287	12.50	18.33	0.9840
	CNN-LSTM-Attention	0.2161	11.08	19.92	0.9859
0.7	CNN	0.1898	13.13	17.60	0.9818
	CNN-LSTM	0.2579	12.59	18.29	0.9822
	CNN-LSTM-Attention	0.2232	11.88	18.71	0.9854
0.8	CNN	0.2194	15.17	16.4	0.9797
	CNN-LSTM	0.2390	13.66	17.67	0.9834
	CNN-LSTM-Attention	0.2355	11.26	19.14	0.9846
0.9	CNN	0.3303	16.51	16.22	0.9720
	CNN-LSTM	0.2739	17.60	15.68	0.9813
	CNN-LSTM-Attention	0.2181	15.08	16.40	0.9857

**Table 3:** Comparison of average metrics with traditional methods.

Metrics	OMP	BP	CNN	CNN-LSTM	CNN-LSTM-Attention
Average RMSE (mV)	0.38	0.21	0.24	0.27	0.23
Average SNR (dB)	5.92	10.26	16.40	17.66	18.33
Average PRD (%)	57.12	38.82	16.72	14.01	12.95

## Data Availability

The original data presented in the study are openly available in PhysioNet at https://doi.org/10.13026/C2F305 (accessed on 8 April 2026).
